# Overcome social anxiety disorder and develop visionary leadership in uncertain environments: The important role of psychological resilience

**DOI:** 10.3389/fpsyg.2022.1106993

**Published:** 2023-01-10

**Authors:** Yi Guan, Yao Wang, Jiaojiao Zhang, Yuan Cao

**Affiliations:** ^1^School of Business, Renmin University of China, Beijing, China; ^2^School of Business, China University of Political Science and Law, Beijing, China; ^3^College of Business, Shanghai University of Finance and Economics, Shanghai, China

**Keywords:** environmental uncertainty, psychological resilience, intrinsic motivation, visionary leadership, social anxiety disorder

## Abstract

Uncertainty is the main feature of the business environment in the post-coronavirus disease 2019 (COVID-19) era. People taking leadership positions in an uncertain environment constantly encounter unprecedented risks and challenges. Many of them have difficulties adapting to such an environment and thus experience severe anxiety, showing the symptoms of social anxiety disorder (SAD), failing to exert effective leadership in social interaction contexts. How can leaders overcome SAD and effectively motivate their subordinates in an uncertain environment? This study explores the important role of psychological resilience. Using sample data collected from 82 leaders and 363 subordinates of eight enterprises in China from May to June 2020, the current study reveals that a high degree of psychological resilience enables leaders to maintain intrinsic motivated at work in an uncertain environment. Leaders with a high degree of psychological resilience perform better in identifying the organizations’ vision and displaying visionary leadership than those with a low degree. The current study enriches the knowledge of leaders’ psychological well-being and effective leadership in the post-COVID-19 era.

## 1. Introduction

Vision is the ideal state an enterprise aspires to achieve in the future (Guinot and Chiva, 2019; Ateş et al., 2020). Visionary leadership refers to a leadership style in which leaders communicate their vision to motivate employees in oral or written forms (Stam et al., 2010a; Van Knippenberg and Stam, 2014). In today’s highly dynamic business environment, leaders must balance leading organizational development and meeting the needs of employees (Lu et al., 2020). Visionary leadership provides an effective solution to this challenge. At the organizational level, visionary leadership focuses on identifying key environmental factors, discovering potential opportunities, and creatively constructing enterprises’ future development blueprints (Hobbs, 2010; Kehr et al., 2022). At the interpersonal level, visionary leadership focuses on communicating visions to make subordinates identify that the vision is valuable and could be realized (Stam et al., 2010b).

With the ongoing trend of economic globalization, particularly influenced by the global crisis of coronavirus disease 2019 (COVID-19), the current business environment (e.g., domestic market, government policy, and international trade) is highly dynamic and full of uncertainty, which posed an unprecedented risk for organizations. Many had to face unpredictable demand fluctuations, regulatory changes, disrupted supply chains, and business shutdowns (Chung, 2022; Lian et al., 2022). However, the risk is always together with chance. In an uncertain environment, novel practices can emerge, organizational forms can emerge and die, status orders can be restructured, and rules of engagement can be redefined (Lounsbury, 2002). In such an environment, enterprises face a high degree of challenge and great opportunities for change; therefore, leaders’ vision should facilitate organizational transformation. In addition, employees tend to feel a high degree of anxiety and lack of assuredness in an uncertain environment. Thus, their leaders’ communications of vision need to reduce their stress by showing how uncertainty can be turned into a vision of opportunity and success (Waldman et al., 2001). From this perspective, visionary leadership is of great importance in today’s highly uncertain environment.

However, research on how environmental uncertainty affects visionary leadership is insufficient. At present, few studies have explored the impact of leaders’ value orientation (De Luque et al., 2008), cognitive processes (Shipman et al., 2010), and organizational size (Berson et al., 2001) on the emergence of visionary leadership. An in-depth discussion of the relationship between environmental uncertainty and visionary leadership is lacking. Thus, theoretical analysis and empirical tests on the mechanism and conditions of environmental uncertainty affecting visionary leadership should be conducted to develop leadership research, provide insights for cultivating visionary leadership, and help enterprises better adapt to the uncertain business environment.

Self-determination theory serves as a key theoretical perspective for studying the relationship between environmental uncertainty and visionary leadership. Self-determination theory provides a framework for the relationships among the environment, motivation, and behavior. The theory holds that environmental factors and personal traits jointly affect people’s motivation, which in turn affects their behavior (Ryan and Deci, 2017). From the perspective of the self-determination theory, uncertainty in the working environment may shape leaders’ visionary leadership behavior through their motivation. This process may be moderated by some personal characteristics of leaders. Based on self-determination theory, the current study proposes a moderated mediation model and explains the mechanism of environmental uncertainty influencing visionary leadership in detail. The study also reveals the important personal characteristics of leaders that moderate this process and tests the theoretical hypothesis by collecting data from multiple time points and sources.

Our study contributes to visionary leadership research in three ways: (1) We highlight the bright side of environmental uncertainty and expand the antecedent research of visionary leadership by examining the association between environmental uncertainty and visionary leadership. (2) Based on the self-determination theory, we elaborate on the psychological mechanism of environmental uncertainty influencing visionary leadership. (3) We elaborate on how environmental uncertainty and leaders’ psychological resilience interact and highlight high psychological resilience as the boundary condition under which environmental uncertainty boosts leaders’ intrinsic motivation and visionary leadership.

The remainder of this paper is organized as follows. “Introduction” reviews the existing literature and proposes research hypotheses. “Literature review and hypothesis development” describes the data sources and research methods used in this study. “Methodology” presents the hypotheses and analysis results. Then, “Result analysis” discusses the results of the study, analyzes its related theoretical significance, and proposes specific measures for intervention in leadership behaviors from the perspectives of environmental uncertainty and leaders’ characteristics. “Discussion” summarizes the conclusions and limitations of the study and proposes a research outlook for future studies.

## 2. Literature review and hypothesis development

### 2.1. Self-determination theory

Self-determination theory is a motivation theory proposed by Deci and Ryan (1985). This theory is particularly concerned with how social-contextual factors support or thwart people’s prosperity by satisfying their basic psychological needs. Based on the principle of organic philosophy, this theory holds that humans are born with the potential for self-development and that those who can realize their potential will show strong cognitive ability, creativity, and proactivity. Based on the principle of dialectical philosophy, the theory also points out that, although people’s tendency toward self-development is innate, the development of this process is affected by the external environment (Ryan and Deci, 2017).

The self-determination theory elaborates on how the external environment affects individual motivation and behavior. The theory identifies three basic psychological needs of human beings, namely, needs for autonomy, competence, and relatedness. Specifically, the need for autonomy refers to the individual’s pursuit of the emerging experience of being the initiator in causal relationships and plays a dominant role in activities. The need for competence refers to the desire to display one’s capability and interact effectively with the social context. The need for relatedness refers to the pursuit of interpersonal acceptance and belongingness (Deci and Ryan, 2000).

Activities that enable people to seek out novelty and challenges, explore new environments, and undertake new adventures can satisfy individuals’ basic psychological needs for autonomy and competence, stimulating intrinsic motivation and driving people to show greater cognitive ability, creativity, and proactivity to achieve self-development. By contrast, environmental factors that cannot meet basic psychological needs may hinder individuals’ intrinsic motivation and overall self-development.

### 2.2. Environmental uncertainty and visionary leadership

Environmental uncertainty refers to the unpredictability of the context or organizational variables that affect corporate performance (Xu et al., 2021). Although an uncertain environment imposes high job demands on company leaders, in this study we propose that it also creates favorable conditions for the emergence of visionary leadership.

On the one hand, an uncertain environment contains many potential opportunities and risks, whereby a few decisions made by leaders can have a tremendous impact on enterprise survival (Waldman et al., 2001). Self-determination theory points out that the experience of making change and responsibility satisfies people’s need for autonomy (Deci and Ryan, 1985). Therefore, with the increase in environmental uncertainty, leaders would strongly feel their importance to enterprise development, motivating them to invest more time and energy in analyzing situational variables, identifying opportunities, and proposing inspiring visions.

On the other hand, leaders need to develop feasible strategies despite the constraints of environmental uncertainty and rebuild employees’ confidence in themselves and the future of the enterprise (Waldman et al., 2001). Self-determination theory claims that an optimal challenge is beneficial for individuals to reach their full potential and obtain a sense of achievement, which is necessary for satisfying the needs of competence. Simple tasks, although easy to accomplish, are boring in nature and cause dissatisfaction with the need for competence (Ryan and Deci, 2017). Therefore, compared with a stable environment, an environment full of uncertainty is more likely to stimulate leaders to fully mobilize their abilities and enthusiasm, motivating subordinates through the construction and expression of inspiring visions (Waldman et al., 2001, 2004). Based on the above analysis, this study proposes the following hypothesis:

*H1*: Environmental uncertainty is positively correlated with visionary leadership.

### 2.3. Mediating role of leader intrinsic motivation

As mentioned earlier, an uncertain working environment would satisfy leaders’ needs for autonomy and competence, thereby creating favorable conditions for visionary leadership. However, satisfying basic psychological needs does not directly influence people’s behavior. According to the self-determination theory, the satisfaction of basic psychological needs first stimulates intrinsic motivation, which subsequently drives behavior (Ryan and Deci, 2017).

Intrinsic motivation refers to the inner motive force inspired by one’s interests and enjoyment of the activity (Ryan and Deci, 2000). A working environment that satisfies basic psychological needs promotes people’s self-development; such a nourishing experience would inspire people’s interest in their work content and therefore become more engaged in work activities (Ryan and Deci, 2017). For business leaders, uncertain environments provide good opportunities to exert strong leadership. In strategizing and inspiring subordinates, the experience of autonomy and competence stimulates leaders’ intrinsic motivation.

When driven by intrinsic motivations, individuals will actively search for information related to tasks and proactively know about their working environment (Ryan and Deci, 2017). Through in-depth processing of information in the environment, leaders can conceive organizational visions that benefit long-running organizational development and are both challenging and feasible (Strange and Mumford, 2005; Watts et al., 2019). Meanwhile, under intrinsic motivations, leaders will increase their confidence in their working abilities, believe that they can master the processes and results of work, and have more courage to bear risks and express challenging organizational visions (Sy et al., 2018). Intrinsic motivations will also drive leaders to convey organizational visions to subordinates sincerely and fully passionately, inspiring them to believe that the visions these leaders propose can be realized (Malik et al., 2021). Based on the above analysis, this study proposes the following hypothesis:

*H2*: A leader’s intrinsic motivation mediates the relationship between environmental uncertainty and visionary leadership.

### 2.4. Moderating role of leader psychological resilience

Although environmental uncertainty creates favorable conditions for the emergence of visionary leadership, not all enterprise leaders can show a high level of visionary leadership style when dealing with an uncertain business environment. Self-determination theory holds that the level of an individual’s intrinsic motivation is not only affected by the external environment but also depends on the degree to which an individual’s ability matches it (Deci, 1975; Deci and Ryan, 1980). Following this argument, the relationship between environmental uncertainty and leaders’ intrinsic motivation may be affected by their abilities.

Psychological resilience is the personal ability to maintain a positive mental state in stressful situations (Elkington et al., 2017). Prior studies suggested that individuals with a high level of psychological resilience are more likely to view adversity as a challenge that helps to improve their performance. Therefore, they tend to proactively regulate their emotion and solve problems. By contrast, individuals with a low level of psychological resilience tend to view adversities as hindrances that threaten their performance. In this situation, they are more likely to suffer from negative emotions (e.g., anxiety, depression) and therefore cannot focus on problem-solving (Fisher et al., 2019). Evidence from work settings suggests that individuals with high psychological resilience can successfully adapt to adverse situations. Moreover, some individuals show growth and positive changes after advertising (Britt et al., 2016; Hartmann et al., 2020).

Uncertain environments consist of numerous variables and contingencies, imposing considerable workloads on leaders. In uncertain work settings, understanding the direction in which an environment might be changing, the potential impact of those changes on that individual organization, and whether or not particular responses to the environment might be successful is difficult for leaders (Milliken, 1987). Taking the catering industry as an example, during the COVID-19 epidemic, predicting when they have to stop their business in response to the zero COVID policy in China is nearly impossible for managers. Therefore, managers were faced with so many dilemmas, such as whether or not to purchase fresh ingredients, start takeout services, and cut staff.

Evidently, an environment perceived in such a manner would tend to generate a high degree of stress on the part of an organization’s leaders. Psychological resilience therefore plays an important role in leaders’ adaptation to an uncertain environment. Specifically, leaders with high psychological resilience tend to focus on the bright side of an uncertain environment. For example, Waldman et al. (2001) suggested that in uncertain environments, leaders could have more influence on their subordinates. Khoiri (2020) suggested that in an uncertain environment, visionary leadership can facilitate organizational change. By contrast, leaders with low psychological resilience tend to focus on the dark side of an uncertain environment. For example, in an uncertain environment, a few erroneous decisions could result in severe trouble and possibly risk the survival of the organization (Waldman et al., 2001).

Elkington et al. (2017) pointed out that, in an environment full of uncertainties, leaders with high psychological resilience can optimistically treat challenges that appear one after another, proactively seize fleeting opportunities, and actively find ways to overcome different difficulties and obstacles in addition to realizing personal improvement in wisdom and capabilities during this process. Those with low psychological resilience cannot withstand stress in an environment full of uncertainties, and they always feel helpless and lost, thereby losing confidence and motivation in leadership. Therefore, we propose that the indirect effect of environmental uncertainty on visionary leadership is influenced by leaders’ psychological resilience. For leaders with higher psychological resilience, their psychological needs of autonomy and competence can be satisfied by effectively coping with the stress and challenges that environmental uncertainty brings, which further enhances their intrinsic motivation and boosts them to display visionary leadership. For leaders with lower psychological resilience, however, their lack of ability to cope with uncertain environmental challenges will prevent them from satisfying the basic psychological needs of autonomy and competence. Therefore, environmental uncertainty does not enhance the intrinsic motivation of low psychological leaders. From the above analysis, this study proposes the following hypothesis:

*H3*: A leader’s psychological resilience moderates the indirect, positive relationship between environmental uncertainty and visionary leadership. When the leader’s psychological resilience is higher, the indirect positive relationship between environmental uncertainty and visionary leadership is stronger.

**Figure 1:**
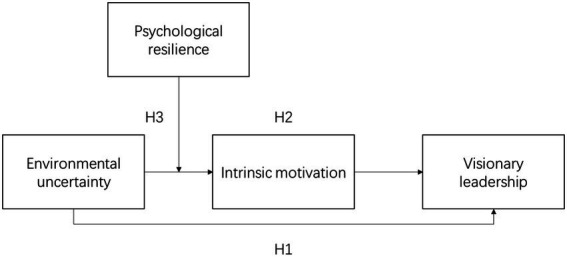
Theoretical hypothesis model of this study.

According to the above analysis, this study constructs a model demonstrating the relationship among environmental uncertainty, intrinsic motivation, visionary leadership, and psychological resilience, as shown in Figure 1.

## 3. Methodology

### 3.1. Data source

First, survey samples were formed through a non-probability sampling mode that combines convenience and snowball sampling. Data were collected using a questionnaire. The survey was conducted on eight state-owned enterprises in Beijing, Hubei Province, Shandong Province, and Hainan Province in China from May to June 2020. These enterprises include agricultural and energy sources. In particular, we contacted the heads of eight enterprises. In turn, they contacted departmental leaders in their organizations, who notified all members of the departmental staff to join the survey. Based on an introduction to study purposes, values, and confidentiality principles and on the consent of participants, an online questionnaire link was sent to participants through WeChat twice during the study period. The first questionnaire was distributed (T1) to measure environmental uncertainty, the leader’s psychological resilience, and the leader’s intrinsic motivation. A month later, the second questionnaire was distributed (T2) to measure visionary leadership.

In this study, 82 departmental leaders and 363 immediate subordinates were recruited, resulting in 445 survey participants (on average, each leader had 4.43 subordinates). All leaders participating in the survey were medium-and high-level management personnel, such as department managers and office directors, with a management range of three to six subordinates. Among all the survey participants, male leaders accounted for 77.0%, female leaders accounted for 23.0%, male employees accounted for 62.7%, and female employees accounted for 37.3%. Regarding the age distribution of leaders, those who were 18–25 years old accounted for 0, 1.2% for 25–30 years old, 26.3% for 31–40 years old, 49.0% for 41–50 years old, 23.6% for 51–60 years old, and 0% for over 60 years old. As for the age distribution of employees, those who were 18–25 years old accounted for 4.2, 21.8% for 25–30 years old, 44.8% for 31–40 years old, 21.8% for 41–50 years old, 7.1% for 51–60 years old, and 0.3% for over 60 years old. On average, leaders and employees had worked in the companies for 15.9 and 9.2 years, respectively. On average, leaders and employees worked together for 3.7 years. All survey participants had undergraduate or higher education levels. The effective recovery rates of the two questionnaire surveys were 85.4 and 82.1%. Nine departments had missing values as their leaders or subordinates failed to complete the results. Thus, data from 73 departments were input into the hypothesis-test program. Table 1 describes the basic profile of the sample.

**Table 1:** Sample description.

Variable and category	Statistic
Position	Leader: 82 (18%)
	Subordinate: 363 (82%)
Gender	Male-leader: 63 (77.0%)
	Female-leader: 19 (23%)
	Male-subordinate: 228 (62.7%)
	Female-subordinate: 135 (37.3%)
Age (years)	Leader 18–25: 4.2%
	Leader 25–30: 21.8%
	Leader 31–40: 26.3%
	Leader 41–50: 49.3%
	Leader 51–60: 23.6%
	Leader >60: 0%
	Subordinate 18–25: 0%
	Subordinate 25–30: 1.2%
	Subordinate 31–40: 44.8%
	Subordinate 41–50: 21.8%
	Subordinate 51–60: 7.1%
	Subordinate >60: 0%

Created by the authors.

### 3.2. Variables

The scales adopted in this study were mainly mature scales that are frequently quoted. We first used the standard translation/back-translation method to ensure the reliability and validity of the measurement with these scales in the Chinese context (Brislin, 1980). Before formal issuance, questionnaires were issued to the person in charge of each enterprise for browsing so that the contents of the questionnaire could satisfy enterprise situations. In addition, item descriptions were clear and easy to understand. All the scales applied a five-point scoring system, ranging from “1—very strongly disagree” to “5—strongly agree.” The specific scales used were as follows:

#### 3.2.1. Dependent variable: Visionary leadership

Subordinates were asked to assess their leaders’ visionary leadership behavior in T2. An average score was then obtained through aggregation, reflecting the overall level of the leader’s visionary leadership. The three-item scale developed by Rafferty and Griffin (2004) was used for the questionnaire. These items are “my leader has a clear understanding of where we are going,” “my leader has a clear sense of where he/she wants our unit to be in 5 years,” and “my leader has no idea where the organization is going (R).”

#### 3.2.2. Independent variable: Environmental uncertainty

Subordinates were asked to assess the uncertainty of the working environment in T1. The average score was obtained through aggregation, which reflects the overall level of working environmental uncertainty. We adapted three items from the scale developed by De Hoogh et al. (2005). These items are “my work environment is full of change,” “my work environment is highly dynamic,” and “my work environment offers great opportunities for change.”

#### 3.2.3. Mediating variable: Leader’s intrinsic motivation

As requested in the study, departmental leaders self-report their intrinsic motivations’ level in T1. We adapted four items from the scale developed by Grant (2008). These items are “I enjoy the work itself,” “my work is fun.” “I find the work engaging,” and “I enjoy my work.”

#### 3.2.4. Moderation variable: Leader’s psychological resilience

As requested, departmental leaders conducted a self-report of psychological resilience in T1. This study used the scale developed by Luthans et al. (2007). The scale contained six items. These items are “When I have a setback at work, I have trouble recovering from it, moving on. (R),” “I usually manage difficulties one way or another at work,” “I can be on my own, so to speak, at work if I have to,” “I usually take stressful things at work in stride,” “I can get through difficult times at work because I’ve experienced difficulty before,” and “I feel I can handle many things at a time at this job.”

## 4. Result analysis

### 4.1. Aggregation analysis

Before the hypothesis test, we first aggregated the environmental uncertainty and visionary leadership that the subordinates assessed to the departmental-level variable. During aggregation, we assessed three common indexes in the multi-level analysis: in-group consistency Rwg, in-group correlation ICC1, and in-group correlation ICC2. As shown in Table 2, the average environmental uncertainty Rwg value was 0.87, with ICC (1) = 0.13, ICC (2) = 0.39, *F* (73, 229) = 1.65, and *p* < 0.01; the average visionary leadership Rwg value was 0.94, with ICC(1) = 0.16, ICC(2) = 0.45, *F*(73, 229) = 1.83, and *p* < 0.01.

**Table 2:** Information on the aggregation analysis.

Construct	ICC (1)	ICC (2)	*F*	Rwg
1. Visionary leadership	0.13	0.39	1.65^**^	0.87
2. Environmental uncertainty	0.16	0.45	1.83^**^	0.94

*N* = 73; ^**^p < 0.01. Created by the authors.

### 4.2. Analysis of reliability and validity

In this study, Cronbach’s α was used to measure the internal consistency reliability of the scale. Cronbach’s α coefficient was 0.99 for visionary leadership, 0.88 for environmental uncertainty, 0.93 for the leader’s intrinsic motivation, and 0.86 for the leader’s psychological resilience. As shown in the analysis results, Cronbach’s α coefficient of each scale ranged from 0.86 to 0.99 (Table 3). All values exceeded the minimum acceptable level of 0.7, indicating that the measurement scales used in this study had high internal consistency reliability (Taber, 2018).

**Table 3:** Evaluation of the measurement model.

Construct	Cronbach’s alpha
1. Visionary leadership	0.99
2. Environmental uncertainty	0.88
3. Intrinsic motivation	0.93
4. Psychological resilience	0.86

*N* = 73. Created by the authors.

Mplus 7.11 was used in the study to conduct confirmatory factor analysis on environmental uncertainty, the leader’s psychological resilience, the leader’s intrinsic motivation, and visionary leadership to assess the discrimination validity of the measurement of each research variable. Table 4 presents the results. As shown in Table 4, compared with other alternative models, the fitting index of the four-factor model was good: *x*^2^/*df* = 1.51, CFI = 0.96, TLI = 0.945, RMSEA = 0.08, SRMR = 0.08; CFI and TLI were larger than 0.9; RMSEA and SRMR were larger than 0.08; the fitting indices of the three-factor and two-factor models did not reach the targeted statistical standards. These results indicate that the measurement of the four variables in the research model had good discrimination validity (Hu and Bentler, 1999).

**Table 4:** Discrimination validity of main variables.

Model	Factor	*x*^2^/*df*	RMSEA	SRMR	CFI	TLI
Four-factor model	A, B, C, D	1.51	0.08	0.08	0.96	0.95
Three-factor model	A + B, C, D	4.23	0.20	0.24	0.71	0.65
Three-factor model	A, B + C, D	2.22	0.12	0.09	0.89	0.87
Three-factor model	A, B, C + D	6.73	0.27	0.20	0.49	0.39
Two-factor model	A + B + C, D	5.31	0.23	0.26	0.61	0.54
Two-factor model	A, B + C + D	7.27	0.28	0.21	0.43	0.33

A denotes environmental uncertainty; B denotes a leader’s psychological resilience; C denotes a leader’s intrinsic motivation; D denotes visionary leadership; “+” denotes a combination of former and latter variables. Created by the authors.

### 4.3. Descriptive statistics

Based on the results of the descriptive statistical analysis using SPSS 20.0, Table 5 provides the mean, standard deviation, and correlation coefficient for each research variable. The results show that the correlation coefficient between environmental uncertainty and the leader’s intrinsic motivation was 0.23 (*p* < 0.05), and the correlation coefficient between the leader’s psychological resilience and the leader’s intrinsic motivation was 0.61 (*p* < 0.01). Moreover, the correlation coefficient between the leader’s intrinsic motivation and visionary leadership was 0.19 (*p* < 0.10), and the correlation coefficient between environmental uncertainty and visionary leadership was 0.31 (*p* < 0.05).

**Table 5:** Table of descriptive statistics and correlation coefficients of main variables.

Variable	*M*	SD	1	2	3	4
1. Environmental uncertainty	3.44	0.48	(0.88)			
2. Psychological resilience	3.82	0.55	0.15	(0.86)		
3. Intrinsic motivation	3.51	0.65	0.23^*^	0.61^**^	(0.93)	
4. Visionary leadership	3.91	0.35	0.31^*^	0.13	0.19^+^	(0.99)

*N* = 73; number in brackets on the diagonal indicates the internal consistency coefficient Cronbach’s alpha; **p < 0.01, *p < 0.05, ^+^*p* < 0.10. Created by the authors.

### 4.4. Hypothesis test

In this study, Mplus 7.11 was used for path analysis to test the hypothesis. As Hypothesis 1 proposes, environmental uncertainty is positively associated with visionary leadership. We first analyzed the model without the mediator. The results indicate that the relationship between environmental uncertainty and visionary leadership is significant (*b* = 0.26, *p* < 0.05). Hence, Hypothesis 1 is supported.

The mediation hypothesis was tested *via* the Monte Carlo simulation procedure suggested by MacKinnon et al. (2004). The results from the mediation model indicate that the relationships between environmental uncertainty and intrinsic motivation (*b* = 0.31, *p* < 0.05) and between intrinsic motivation and visionary leadership (*b* = 0.20, *p* < 0.05) are significant. With 20,000 Monte Carlo replications, we found that through the intermediation of leaders’ intrinsic motivation, environmental uncertainty has a significant and positive indirect influence on visionary leadership (indirect effect amount is 0.08, and the 95% confidence interval is [0.00, 0.11]). Hence, Hypothesis 2 is supported.

**Figure 2:**
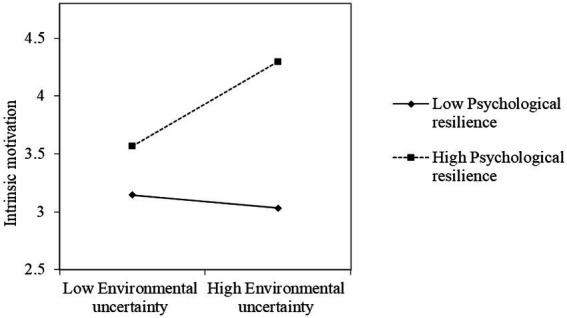
Moderation of the leader’s psychological resilience in the relationship between environmental uncertainty and leaders’ intrinsic motivation.

We analyzed the moderated mediation model to test Hypothesis 3. Table 6 shows the results. We tested the moderating effect of leaders’ psychological resilience on the relationship between environmental uncertainty and leaders’ intrinsic motivation. As shown in the test results, leaders’ psychological resilience plays a significant moderating role in the relationship between environmental uncertainty and intrinsic motivation (*b* = 0.55, *p* < 0.05). Figure 2 shows further simple-effect testing results. When the leader’s psychological resilience is high (+ 1SD), a significant positive correlation exists between environmental uncertainty and the leader’s intrinsic motivation (*b* = 0.51, *p* < 0.05). However, when the leader’s psychological resilience is low (− 1SD), the relationship between environmental uncertainty and the leader’s intrinsic motivation is non-significant (*b* = −0.09, *p* > 0.1).

**Table 6:** Results of full model-based path analysis.

Predictive variable	Dependent variable	
	Intrinsic motivation	Visionary leadership
	*b*	*b*
1. Environmental uncertainty	0.21^*^	0.09
2. Psychological resilience	0.76^**^	−0.12
3. Intrinsic motivation	–	0.22^*^
4. Environmental uncertainty × psychological resilience	0.55^*^	–

*N* = 73; **p < 0.01, *p < 0.05. Created by the authors.

We then tested the moderating effect of leaders’ psychological resilience on the indirect relationship between environmental uncertainty and visionary leadership. We estimated the indirect relationships of environmental uncertainty with visionary leadership *via* intrinsic motivation at higher and lower levels (± 1SD) of psychological resilience using the Monte Carlo simulation method. As shown in Table 7, the leader’s psychological resilience can significantly moderate the indirect relationship between environmental uncertainty and visionary leadership (the effect amount is 0.14, and the 95% confidence interval is [0.08, 0.48]). When leaders’ psychological resilience is high, through the intermediation of leaders’ intrinsic motivation, environmental uncertainty has a significant and positive indirect influence on visionary leadership (indirect effect amount is 0.13, and the 95% confidence interval is [0.06, 0.28]). When the leader’s psychological resilience is low, through the intermediation of leaders’ intrinsic motivation, environmental uncertainty has non-significant indirect influences on visionary leadership (the indirect effect amount is −0.01, and the 95% confidence interval is [−0.24, 0.01]). Hence, Hypothesis 3 is supported.

**Table 7:** Test results of intermediated moderation effects.

Psychological resilience	Conditional indirect effect	95% Confidence interval	
		Lower limit	Upper limit
High	0.13	0.06	0.28
Low	−0.01	−0.24	0.01
Difference	0.14	0.08	0.48

*N* = 73. Created by the authors.

## 5. Discussion

This study uses self-determination theory as the basis, environmental uncertainty as the independent variable, visionary leadership as the dependent variable, leaders’ intrinsic motivation as the mediating variable, and leaders’ psychological resilience as the moderating variable to construct the psychological mechanism model concerning the influences of environmental uncertainty on visionary leadership behaviors. Moreover, these variables were used to analyze the intermediation of leaders and the moderating effects of the leader’s psychological resilience. On this basis, this study proposes specific measures for intervening in leadership behaviors from the perspective of environmental uncertainty and leaders’ characteristics.

First, when the leader’s psychological resilience is high, environmental uncertainty can better stimulate intrinsic motivation, thereby generating visionary leadership behaviors. This result demonstrates the mechanism and boundary conditions for environmental uncertainty to influence visionary leadership, which can enrich research on leadership in an uncertain environment. Several studies focused on the influence of environmental uncertainty on leadership and found that environmental uncertainty can increase uncertainty in leadership courses (Piaskowska and Trojanowski, 2014) and influence leaders’ strategic selection (Justin Tan and Litsschert, 1994). To some extent, these studies illustrated the influence of environmental uncertainty on leadership effectiveness. However, most studies perceived environmental uncertainty as an antecedent or moderation variable, failing to make an in-depth discussion on the mechanism by which environmental uncertainty influences leadership styles. Moreover, few studies focused on how relevant external organizational environments and internal organizational features interact and how they jointly influence leaders (Tan et al., 2016). This study analyzed and verified how environmental uncertainty influences visionary leadership by influencing leaders’ intrinsic motivations. In addition, this study discusses the moderating role of resilience and more comprehensively reflects on the complicated course of how environmental uncertainty influences visionary leadership. Therefore, in practice, enterprises also need to pay attention to the important role played by environmental uncertainty in leaders’ styles. Specifically, enterprises should lead leaders to recognize the challenging and growth factors in an uncertain environment, adopt incentive policies to encourage leaders’ thinking and description of corporate visions and supporting measures in uncertain environments, and use certain strategies to relieve possible negative influences brought about by environmental uncertainty. For example, enterprises can seek to have an accurate understanding of the changes in technologies and markets in the external environment to provide sufficient information for leaders’ objective prediction of the degree of uncertainty of external environments.

Second, the leader’s intrinsic motivation contributes to the improvement of visionary leadership behaviors; when leaders have high psychological resilience, environmental uncertainty may have an indirect positive influence on visionary leadership behaviors by influencing the leader’s intrinsic motivation. Related studies focused on discussing the positive effects of visionary leadership (Kearney et al., 2019; Van der Voet and Steijn, 2021); however, few studies investigated the antecedent variables of visionary leadership. They found that visionary leadership could be influenced by certain factors, such as leadership styles, leaders’ characteristics, and cognitive competence (e.g., Berson et al., 2001; Shipman et al., 2010; Atthirawong et al., 2021). These studies failed to consider how the external environment influences the generation of visionary leadership. Specific environments can influence the development of visionary leadership and the proposition of visions (Fan and Wang, 2017). Based on self-determination theory, this study illustrates that when the leader’s psychological resilience is high, environmental uncertainty and external characteristics will stimulate the leader’s intrinsic motivation, thereby allowing the leader to manifest visionary leadership behaviors. Meanwhile, the leader’s intrinsic motivation had a direct positive effect on visionary leadership. The above findings bring novel theoretical connotations to studying antecedent variables of visionary leadership and enrich existing references and theories. In management practice, enterprises should also pay attention to the positive influences of the leader’s intrinsic motivation on visionary leadership behaviors and adopt corresponding policies to satisfy the leader’s intrinsic motivation. When designing work for leaders, enterprises should give them enough space for independent decision-making, reduce interference in their work, stimulate their passion and interest in work, and enhance their intrinsic motivations to allow them to think about the goals and ideal future of their organizations.

Finally, the leader’s psychological resilience can significantly moderate the relationship between environmental uncertainty and intrinsic motivation. Psychological resilience significantly moderates the indirect relationship between environmental uncertainty and visionary leadership. As shown in the above results, psychological resilience plays an important role in forming visionary leadership in uncertain environments. In previous studies, psychological resilience has been demonstrated in several fields (e.g., clinical and developmental psychology). However, few related studies exist in organizational behavior research at present, in addition to the evident lack of theory-driven empirical research (King et al., 2016). Previous studies focused on the direct positive influence of employees’ psychological resilience (Shin et al., 2012; Malik and Garg, 2020; Santoro et al., 2021). Moreover, only a few have explored the moderating effect of psychological resilience based on perspectives such as stress transactional theory, self-enhancement theory, and resource conservation theory (Kimura et al., 2018; Al-Hawari et al., 2020). Most of the above studies on psychological resilience are based on employees’ perspectives and lack discussion on leaders’ psychological resilience. Researchers called for the study of resilient leadership (Giustiniano et al., 2020). Few studies examined the effects of psychological resilience from the motivational perspective. Based on self-determination theory, from the perspective of motivations, this study enriches the theoretical perspective of psychological resilience research. That is, empirical research results indicate that resilience can help leaders more actively cope with the uncertain environment, enhance intrinsic motivations, and help them adopt visionary leadership behaviors. Given the important role of psychological resilience, enterprises can use corresponding psychological testing to select leaders with psychological resilience to determine and improve ways to cope with complicated and varying environments. Meanwhile, enterprises can adopt certain interventions and guidance to enhance a leader’s psychological resilience, conduct “resilience education,” and help leaders enhance their adaptability to the environment. For example, through training interventions, organizations can make plans to train leaders to cope with complicated events and train leaders’ skills in handling complicated and varying events to enhance their psychological stress resistance and recovery ability.

## 6. Conclusion and implications

### 6.1. Conclusion

Questionnaire survey data from multiple time points and multiple sources from eight enterprises and 73 departments in China were adopted to address the issue of the formation mechanism of visionary leadership. We conducted an empirical analysis of the influence of environmental uncertainty on visionary leadership. Moreover, the intermediation of leaders’ intrinsic motivation and the moderating effects of the leader’s psychological resilience were examined. The following conclusions were drawn: (1) Environmental uncertainty is positively related to visionary leadership. (2) A leader’s intrinsic motivation mediates the relationship between environmental uncertainty and visionary leadership. (3) A leader’s psychological resilience moderates the indirect relationship between environmental uncertainty and visionary leadership. A higher degree of the leader’s psychological resilience promotes the indirect positive effect of environmental uncertainty on visionary leadership *via* the leader’s psychological resilience.

### 6.2. Managerial implications

The above conclusions bring significant insights to corporate management as follows:

1. Intrinsic motivation plays an important role in the occurrence of visionary leadership. Enterprises should give leaders enough rights to self-decision in work design, provide leaders with supportive work environments, let them act according to their circumstances, act when the conditions are the best (use more appropriate people, technology, or equipment), and motivate their intrinsic motivations. Thus, the leaders’ internal motivation is stimulated to think more about the future of the enterprise, prompting them to demonstrate visionary leadership behavior, conceive a vision for the enterprise, and clarify the direction of the enterprise.
2. Enterprises can guide leaders to recognize favorable factors in an uncertain environment and encourage them to think about corporate visions in an uncertain environment. In an uncertain environment, enterprises need to adapt to the external environment through rapid response measures, and to achieve long-term development by strengthening their “immune system” to continuously respond to change. The key is for leaders to quickly adjust their perceptions, accept crises and challenges, and embrace change with the greatest determination to live with change and promote reform and innovation.
3. Psychological resilience is an important feature that leaders exhibit when coping with environmental uncertainty. Thus, enterprises should pay attention to the training and intervention of leaders’ psychological resilience. Particularly when facing an uncertain environment, such as the current global COVID-19 epidemic, leaders with strong psychological resilience are needed to lead the enterprise and help it to overcome the difficulties. This case will help bring long-term growth momentum to the enterprise and enhance the cohesiveness of members within the organization, thereby promoting the long-term development of the enterprise.

### 6.3. Research limitations and future directions

This study provides an in-depth discussion on the mechanism and boundary conditions for environmental uncertainty to influence visionary leadership, contributing to the enrichment of studies on leadership in uncertain environments, remedying deficiencies of studies on visionary leadership antecedent research, and remaining significant to some extent in theory and practice. However, the present study is inevitably limited by the following aspects. (1) The study sample was mainly sourced from state-owned enterprises. Thus, the sample sources were relatively singular. Future studies could collect data from other types of enterprises to verify further the model proposed in this study. (2) The study reveals the effects of environmental and leadership factors on visionary leadership but fails to consider whether subordinates can influence the above relationships. Hence, future studies can discuss how the features and behaviors of subordinates influence the generation of visionary leadership. (3) This study mainly focuses on the positive effects of psychological resilience on leaders’ coping with uncertain environments but neglects the influences of other leader factors on this course. Hence, future research can consider using other features and abilities of leaders as moderation variables. For example, in the case of a prevailing proactive personality, future work can determine whether these leaders are more willing to cope with the challenges of an uncertain environment, enhance their intrinsic motivations, and generate visionary leadership.

## Data availability statement

The original contributions presented in the study are included in the article/supplementary material, further inquiries can be directed to the corresponding author.

## Author contributions

YG contributed to the conception of the study, wrote the first draft of the manuscript, and worked on the coding of tables and figures. YW and JZ contributed to the conception and design of the study. YC helped perform the analysis with constructive discussions. All authors contributed to the article and approved the submitted version.

## Funding

This study was supported by the National Natural Science Foundation of China (No. 72102228).

## Conflict of interest

The authors declare that the research was conducted in the absence of any commercial or financial relationships that could be construed as a potential conflict of interest.

## Publisher’s note

All claims expressed in this article are solely those of the authors and do not necessarily represent those of their affiliated organizations, or those of the publisher, the editors and the reviewers. Any product that may be evaluated in this article, or claim that may be made by its manufacturer, is not guaranteed or endorsed by the publisher.
